# Comparison of erector spinae plane and paravertebral nerve blocks for postoperative analgesia in children after the Nuss procedure: study protocol for a randomized controlled non-inferiority clinical trial

**DOI:** 10.1186/s13063-022-06044-y

**Published:** 2022-02-14

**Authors:** Min Xu, Guangchao Zhang, Jingxuan Gong, Jing Yang

**Affiliations:** 1 Department of Anesthesiology and National Clinical Research Center for Geriatrics, West China Hospital of Sichuan University, Chengdu, Sichuan Province China; 2 Department of Biomedical Engineering, Northwestern University, Evanston, IL USA

**Keywords:** Erector spinae plane block, Thoracic paravertebral block, Pectus excavatum, Pain management, Randomized controlled trial

## Abstract

**Background:**

Thoracic paravertebral block (TPVB) is a widely advocated regional technique for alleviating postoperative pain in children undergoing elective pectus excavatum repair. However, this technique is associated with some undesirable adverse events. Recently, the erector spinae plane block (ESPB) has been introduced as a practical alternative to the TPVB in thoracic surgery. This interfascial regional anesthesia technique interrupts pain sensation by injecting local anesthetics between the muscular layers of the thoracic wall. Several case series described it as an effective pain management technique following pectus excavatum repair. Therefore, this trial is designed to test the hypothesis that ESPB is non-inferior to TPVB in postoperative pain control after pectus excavatum repair.

**Methods:**

This is a prospective randomized double-blind non-inferiority trial. A total of 40 patients aged 4 to 18 years undergoing Nuss surgery will be randomly assigned to receive pain treatment with either ESPB or TPVB. All patients will receive additional systemic multimodal analgesia with an intravenous patient-controlled analgesia pump and acetaminophen. The primary outcome is the pain intensity at rest, 24 h postoperatively. Secondary outcomes include accumulated morphine-equivalent consumption, postoperative pain scores, emergence agitation incidence, time of the first mobilization, time to first rescue analgesia, complications related to pain treatment, and morphine-related adverse events.

**Discussion:**

This will the first randomized controlled trial to compare ESPB with TPVB for analgesia after pectus excavatum repair. This trial aims to provide important clinical evidence to elaborate on the analgesic mechanism of ESPB in children.

**Trial registration:**

ClinicalTrials.govNCT05034601. This trial was prospectively registered.

**Supplementary Information:**

The online version contains supplementary material available at 10.1186/s13063-022-06044-y.

## Background

Pectus excavatum (PE) is the most common congenital chest wall deformity in children and adolescents, with an incidence of approximately 1 in 400 live births [1]. The Nuss procedure, a minimally invasive procedure involving placement of stainless steel or titanium bar underneath the sternum with thoracoscopic guidance, has been widely used to correct this deformity. While the incisions involved in this repair are minimal, intraoperative traction and dissection of intercostal muscles, as well as stretching and pressure on the chest wall, resulting in severe postoperative pain [2]. Adequate analgesia after PE repair is essential for pediatric patients because of its potential benefits in decreasing atelectasis, accelerating rehabilitation, shortening hospital stay, and improving parental satisfaction.

Conventional patient-controlled intravenous analgesia with opioids has been proved to be an effective technique with fewer complications. However, high doses of systemic opioids may increase the incidence of adverse reactions, such as respiratory depression, nausea, and vomiting. Although thoracic epidural analgesia has been advocated as a superior method for controlling pain after PE repair [2], concerns about the invasiveness and safety of these approaches have hindered practitioners from using these techniques routinely in these minimally invasive procedures. Thoracic paravertebral block (TPVB) is utilized as a substitute for epidural analgesia to provide postoperative analgesia for thoracic surgery [3]. However, there is still a risk of pneumothorax and vascular nerve injury. Most patients still experience mild to moderate pain postoperatively, regardless of analgesic strategies [4]. Pain management in pediatric patients undergoing Nuss surgery remains a challenge.

In order to facilitate effective analgesia and minimize the risk of puncturing adjacent structures, anesthesiologists have been trying to find new approaches constantly. Recently, the erector spinae plane block (ESPB) for postoperative regional analgesia has aroused attention. ESPB is an interfascial plane block whereby local anesthetics are injected beneath the iliocostalis, longissimus, and spinalis muscles. It is likely to take effect by local anesthetic diffusing into the paravertebral space, blocking the dorsal and ventral branches of the thoracic spinal nerves covering the dermatome. ESPB is less invasive than TPVB, is relatively simpler to perform, and has been applied successfully in children after thoracic procedures [5]. Several case series described it as an effective pain management technique following pectus excavatum repair. The aim of this double-blinded, randomized, controlled, non-inferiority study is to investigate whether ESPB provides comparable postoperative analgesia to TPVB following Nuss surgery. Our primary hypothesis is that ESPB would provide non-inferior analgesia to TPVB after PE repair in a multimodal analgesic regimen, taking the pain scores at first 24 h as the major outcome.

## Methods

### Trial design and setting

**Fig. 1:**
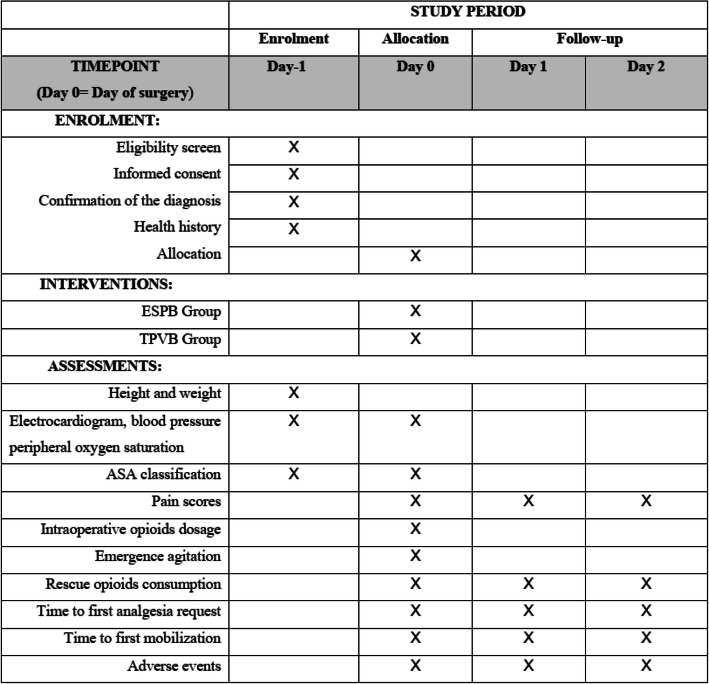
Standard Protocol Items: Recommendations for Interventional Trials (SPIRIT) recommended content for the schedule of enrolment, interventions, and assessments

**Fig. 2:**
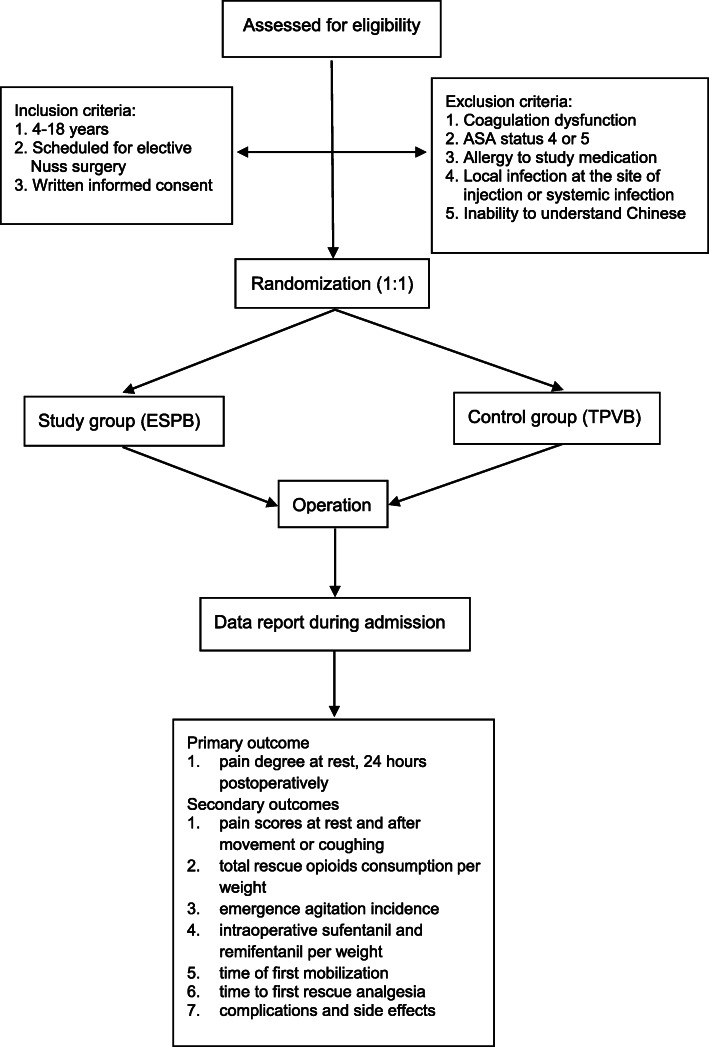
Flowchart of the trial design. ESPB, erector spinae plane block; TPVB, thoracic paravertebral block; ASA, American Society of Anesthesiologists

This prospective, randomized, double-blind, non-inferiority trial will be performed at West China Hospital, China. The protocol is developed according to the Standard Protocol Items: Recommendations for Interventional Trials (SPIRIT) 2013 Statements, Fig. 1 (the SPIRIT checklist is presented in Appendix S1). The Consolidated Standards of Reporting Trials (CONSORT) flow diagram will be followed in reporting the final results of this trial. A flowchart of the trial design is shown in Fig. 2.

### Participant eligibility and consent

Patients diagnosed with PE scheduled for the Nuss procedure at West China Hospital will be screened and recruited for eligibility. Recruitment will be completed at the preoperative interview 1 day prior to surgery by an independent researcher.

Inclusion criteria:

- Age between 4 to 18 years
- Scheduled for elective Nuss surgery
- Written informed consent is obtained from the parents of each patient

Exclusion criteria:

- Coagulation dysfunction
- American Society of Anesthesiologists (ASA) Physical Status 4 or 5
- Allergy to study medication
- Local infection at the site of injection or systemic infection
- Inability to understand Chinese

### Intervention

#### Both groups

After patient arrival in the operating room, electrocardiogram, noninvasive blood pressure, and peripheral oxygen saturation will be monitored throughout surgery. General anesthesia will be induced in both groups with intravenous midazolam 0.05 mg/kg, fentanyl 3 μg/kg, propofol 2.5 mg/kg, and cisatracurium 0.2 mg/kg. After tracheal intubation, general anesthesia will be maintained with sevoflurane at 1–1.5 MAC in both groups. The children will be placed in the lateral decubitus position to receive TPVB or ESPB under aseptic conditions, respectively. Intravenous remifentanil (0.1 μg/kg/min) will be continuously injected for intraoperative analgesia. If necessary, remifentanil infusions will be titrated up to a dose of 0.3 μg/kg/min, maintaining hemodynamic parameters within 20% of baseline values. Intravenous hydromorphone 10 μg/kg and ondansetron hydrochloride 0.1 mg/kg will be injected before incision closure. All patients will receive neostigmine reversing neuromuscular blockade before extubation.

#### TPVB group

A 6- to 12-MHz linear probe (Mindray Anesus ME7, China) will be used to identify the T5 vertebrae and the spinous process, transverse process, and the paravertebral space at the target vertebra level. With the puncture area and the ultrasound probe kept clean and sterile, a 21-gauge 50-mm insulated needle (Uni-Plex Nanoline, Germany) will be inserted into the paravertebral space via an in-plane parasagittal approach. After perforating the costotransverse ligament, 0.25% ropivacaine 0.5 ml/kg will be injected after negative aspiration in 5-ml increments. Anterior movement of the pleura indicated the appropriate spread of the local anesthetics in the paravertebral space. The process will be repeated on the contralateral side.

#### ESPB group

The ESPB will be performed as described by Forero et al. [6]. A 6- to 12-MHz linear probe will be placed in a longitudinal orientation over the T5 transverse process. After identifying the interfascial plane beneath the iliocostalis, longissimus, spinalis, trapezius, and rhomboid major muscles, a 21-gauge 50-mm needle (Uni-Plex Nanoline, Germany) will be inserted into the tissue from caudal to cephalad with an in-plane technique until contacting the transverse process. A bolus of 0.25% ropivacaine 0.5 ml/kg will be injected into the fascial layer after tip positioning was confirmed by careful hydro-dissection with saline. Contralateral ESPB will be performed similarly.

#### Standard post-operative pain treatment

Intravenous patient-controlled analgesia (PCA) pump will connect to all patients before leaving the operating room. Sufentanil 4 μg/kg and granisetron 0.2 mg/kg diluted in 100 ml of normal saline will be used as the PCA infusate. The pump will be programmed to deliver 0.5 ml intravenous bolus on demand, with a lockout interval of 15 min, a background infusion of 1 ml/h, and a maximum dose of 4 ml/h. The standard postoperative analgesic regime consists of 15 mg/kg acetaminophen orally four times daily (maximum dose of 2 g per 24 h), and if needed, or choose instead of the PCA pump, supplemental opioids. The total postoperative opioid consumption will be recorded in case report form (CRF) and the total consumed intravenous opioids dose and the supplemental opioids will be converted to morphine equivalents.

#### Strategies to improve adherence to interventions

Clinical research coordinator is set up to guarantee subject compliance. Surgeons will receive a daily short text message reminding them to prescribe analgesics. During the follow-up period, participants will be reminded to take their medications every day.

#### Relevant concomitant care permitted or prohibited during the trial

Implementing ESPB or TPVB for postoperative analgesia in children after the Nuss procedure will not require alteration to usual care pathways (including use of any additional medication) and these will continue for both trial arms.

### Outcomes

#### Primary outcome

The primary outcome of this study is the pain scores at rest, 24 h postoperatively. Pain cores are registered on a Numeric Rating Scale (NRS) 0–10/10, where 0 = no pain and 10 = worst pain imaginable

#### Secondary outcomes

(A) The pain degree at rest and after movement or coughing (dynamic) will be assessed at 3, 6, 12, 24, and 48 h after surgery, respectively
(B) The total rescue morphine-equivalent consumption per weight at predetermined time intervals (24 and 48 h) after surgery
(C) Emergence agitation will be registered using pediatric anesthesia emergence delirium (PAED) at 5, 15, and 30 min after extubation
(D) Total dosage of intraoperative sufentanil and remifentanil per weight
(E) The time to first analgesia request
(F) The time to first mobilization
(G) Possible block-related side effects such as infection at the injection site, pneumothorax, vascular puncture, and local anesthetic toxicity, as well as adverse events such as postoperative nausea and vomiting (PONV), itching, and dizziness

### Participant timeline

For included participants, enrollment will be performed 1 day prior to surgery and reconfirmed on the day of surgery. Then random allocation will be assigned by a specific statistician before intervention. The accrual period of this trial is expected to be approximately 1 year. The timeline is shown in Fig. 1.

### Sample size calculations

The primary outcome of this non-inferiority trial is the pain scores at rest, 24 h postoperatively. A higher pain score in clinical practice indicates that the patients suffer more severe pain. No non-parametric adjustment was used in the sample size calculation. We used the sample size estimation with the non-inferiority test for the difference between two groups using the PASS 11.0 software. Considering the data from our pilot study (*n*=12, unpublished data between April and June 2021 from the same hospital and the same investigator), the mean pain scores at rest 24 h postoperatively after ESPB was 2.8 and 2.3 of TPVB. Based on previously published data, a minimal clinically significant difference in pain severity was 1.3 [7]. A sample size of 36 is required to provide a power of 0.8 and a one-sided α of 0.025. We decide to recruit 40 patients into the study, given the possibility of dropout.

### Allocation

The study is a double-blinded, randomized, controlled, and non-inferior trial. The SPSS 25.0 software package (SPSS Inc, Chicago, IL, USA) is used to generate random numbers with a block size of 4 by biostatisticians who will not participate in the statistical analysis of the data. When the participants are included in the clinical study, they will receive the modality of treatment, which corresponds to the number in the randomization list. The modality of treatment will be hidden in sequentially numbered, opaque, sealed envelopes, kept by a secretary who is not further involved in the trial. The randomization will follow the final positive acknowledgment on inclusion from the participant.

### Blinding

The participants will be blinded to the allocation. Due to the close proximity of needles for injection of ESPB and TPVB, and the block performed after the implementation of general anesthesia, it is difficult for children and their parents to detect clinical differences. The anesthesiologist performing the nerve block, responsible for intraoperative management, and the surgeons are independent individuals. Investigators not involved in nerve block and intraoperative management are designated to postoperative follow-up. In addition, a trained anesthesiologist designated not to perform the block will objectively assess the clinical characteristics of the block.

### Safety

Security monitoring follows through the study at all times. All the adverse events related to the study intervention will be recorded in the study database and reported as required to the adverse event registration system of West China Hospital within the prescribed period of time, depending on the severity. The clinical trial monitoring committee of West China Hospital decide on a case-by-case basis whether to terminate the study. No efficacy interim analysis is set up in this study.

### Adverse event reporting and harms

Clinically relevant adverse effects as related to treatments will be considered: infection at the injection site, pneumothorax, and local anesthetic poisoning during the trial. Once an adverse event occurs, the investigator shall state to the participant with their parents, and the participant is required to report truthfully the changes in his condition following the treatment. For adverse events occurring during the study period, their symptoms, extent, time of appearance, duration, handling measures, experience, etc. should be recorded on a CRF, evaluated their relevance to the study treatment, and recorded in detail by the investigator, signed, and dated. In the event of a serious adverse event in a study, the project leader must take immediate measures to protect the safety of subjects. The investigator should also report to the ethics committee, the data monitoring committee, and relevant regulatory bodies as required indicating expectedness, seriousness, severity, and causality. When urgent breaking of blinding is required for a serious adverse event to occur in a clinical study, the blinding should be broken jointly by the project leader, investigator, clinical monitor.

### Data collection and management

Preoperative, intraoperative, and postoperative follow-up data will be collected for the proper storage as the study raw materials. All collected data is registered in a CRF, devised for every included patient. All data are entered into the electronic Case Report File, and any traces of entries, modifications, deletions, etc. are retained in the log showing who and when they were changed. Paper version data are input into the electronic Case Report File by two research assistants, and double entry is adopted. The electronic data are backed up, and the backed-up electronic data are saved by another person. The investigator checks the data again according to CRF to ensure the accuracy of the data. A specific biostatistician will complete the screening and randomization of research data.

### Recruitment

Clinical trial information will be presented by posting posters in West China Hospital.

The contact information of the research center and the researchers is found in the posters. At the same time, the researcher also recruits the participants in the outpatient clinic. They evaluate and recruit prospective candidates by introducing the trial to them.

### Participant retention and withdrawal

All reasonable efforts will be made to ensure optimum participant engagement and to reduce study attrition. The follow-up of participants is managed by the clinical research coordinator. Before joining the project, the patient is promised to receive further rehabilitation therapy guidance from a specialist after the trial. All participants will have the right to withdraw from the study at any stage. If the patients decided to do so, which will not harm the relationship with the investigator, they will continue to receive the best available treatment. Participants who discontinue the treatment during the trial will be encouraged to complete all visits as scheduled. On the consent form, participants will be asked if they agree to use of their data should they choose to withdraw from the trial. If the participant is willing to provide them, any data already collected from that participant will be analyzed separately before uncovering blindness and finally are explained separately in the outcome part of this trial.

### Frequency and plans for auditing trial conduct

The project management team meet biweekly to ensure that the study is being conducted in accordance with the study protocol. And the data monitoring and ethics committee of West China Hospital meet every 3 months to ensure the safety of the study.

### Additional consent provisions for collection and use of participant data and biological specimens

Participants will be asked for permission for the research team to share relevant data with people from the Universities taking part in the research or from regulatory authorities, where relevant. This trial does not involve collecting biological specimens for storage.

### Statistical methods

The normality of the continuous data was first assessed with the Shapiro-Wilk test. Normally and non-normally distributed variables will be described by the mean (standard deviation (SD)) or median (interquartile range (IQR)). Between-group differences were evaluated using the independent Student’s *t*-test for continuous normally variables, whereas non-parametric data will be compared using the Mann-Whitney *U* test, as appropriate. A chi-square test or Fisher’s exact test will be used for categorical variables. For the non-inferiority evaluation, we calculated the 95% CIs of the differences in pain scores (based on TPVB minus ESPB). If the upper bound of the one-sided 95% CI is smaller than 1.3, we will conclude that the pain score at postoperative 24 h in the ESPB group is non-inferior to that in the TPVB group. Repeated-measures analysis of variance was carried out within groups for variables with normal distribution. For data with nonnormal distribution, the Friedman test was used for repeated measures. For all these analyses, a two-tailed *P* value less than 0.05 was considered to be statistically significant. There will be no subgroup analyses, for instance, based on gender. Any change of the plan for statistical analysis will be described in the following publication.

### Ethical approvals

The study was approved by the Ethics Committee of the West China Hospital (Approval-No: 2021-866, protocol version 1.0). The trial will be conducted in accordance with the Declaration of Helsinki, as well as the International Conference on Harmonization (ICH). Collected data will not be linked to any individual or personal identifiers. Written informed consent to participate will be obtained from all participants and their parents (the ethics committee of West China hospital requires that subjects under 18 years old must obtain informed consent from their parents before being included in the study).

### Protocol amendments

Any change in the study protocol will require an amendment. Any proposed protocol amendments will be initiated by the principal investigators. All revised versions of the protocol will be signed by the staff, and the amendment forms will be submitted to the Ethics Committee for approval. Any deviations from the protocol are fully documented using a breach report form. The protocol also is updated in the clinical trial registry.

### Composition of the data monitoring committee, its role, and reporting structure

The data monitoring committee is set up by the West China Hospital, and its members have no interest relationship with the sponsor. The committee monitors the eligibility, integrity, safety, and effectiveness of data through system and question the problem data.

### Data retention

To enable evaluations and audits from regulatory authorities, data obtained from participants will retained confidential and stored securely at the Department of Anesthesiology, West China Hospital, Sichuan University for at least 5 years. The investigators will keep records including the identity of all participants, all original signed informed consents, serious adverse event recordings, and CRF. The data will be kept securely and not revealed to other people without appropriate permission.

### Provisions for post-trial care

There is no anticipated harm and compensation for trial participation. After the trial, patients will be provided with guidance on specialized rehabilitation treatment protocols.

### Dissemination plans

Information about the trial is published at ClinicalTrials.gov. The outcomes of the study, whether positive or negative, will be disseminated in an international peer-reviewed medical journal.

### Plans to give access to the full protocol, participant-level data, and statistical code

This study protocol is registered with the registration number NCT05034601 in ClinicalTrials.gov (https://clinicaltrials.gov/ct2/show/NCT05034601). The datasets analyzed during the current study are available from the corresponding author on reasonable request.

## Discussion

The ESPB is a novel fascial plane block recently introduced into clinical practice with the advantage of being relatively safe and convenient to perform. Currently, the mechanism of action of ESPB is not yet fully understood. Local anesthetic diffusion to the thoracic paravertebral space may be the primary mechanism of ESPB, although remains controversial. An updated review summarized that evidence of paravertebral dye penetration was found in 12 of 16 cadaveric studies with thoracic ESPB [8]. With the publication of clinical studies and meta-analyses, the promising effect of ESPB for pain management after abdominal and thoracic surgery was demonstrated [8]. This prompted us to speculate that ESPB may have a similar analgesic profile to TPVB and in turn to conduct a trial to compare the two techniques.

As anesthesiologists, the safety and necessity to implement nerve blocks must be carefully assessed by analyzing risks and benefits. In our center, the ultrasound guidance in-plane technique of TPVB is usually performed by an experienced anesthesiologist, to minimize the potential risks. Therefore, we designed this study, to evaluate whether the ESPB with operation simplicity could be an appropriate alternative, especially in the setting of multimodal therapy. The strengths of the trial design are its randomization, blinding, and noninferiority test. It is designed as a noninferiority trial as the analgesic efficacy of successful TPVB is evident. Furthermore, it is not necessary to demonstrate the analgesic advantage of the ESPB, because it is superior to TPVB in terms of feasibility and safety. We hypothesized that analgesia would be non-inferior 24 h postoperatively as measured on a pain degree with ESPB. To our knowledge, this is the first trial to assess whether ESPB provides sufficient and effective analgesia in Nuss operation. The expected results will provide clinical evidence to verify the analgesic mechanism of ESPB and promote its application in PE repair. Furthermore, if our trial yields positive results, there is potential that ESPB could be recommended as an alternative block for postoperative analgesia in Nuss surgery, to circumvent TPVB which carries risks of failure, vascular puncture, and pneumothorax.

## Trial status

At the time of manuscript submission, the study had been launched and a few patients had participated in the trial. The recruitment was beginning on 25 September 2021. Inclusion will continue until 40 patients with useful data have been included in the trial and is expected to be completed in September 2022.

## Supplementary Information

**Additional file 1.** SPIRIT 2013 Checklist: Recommended items to address in a clinical trial protocol and related documents*.

## Data Availability

All investigators will have access to the final de-identified study dataset from the corresponding author for the purpose of scientific publications.

## Abbreviations

TPVB: Thoracic paravertebral block
ESPB: Erector spinae plane block
PE: Pectus excavatum
SPIRIT: Standard Protocol Items: Recommendations for Interventional Trials
CONSORT: Consolidated Standards of Reporting Trials
ASA: American Society of Anesthesiologists
PCA: Patient-controlled analgesia
CRF: Case report form
NRS: Numeric Rating Scale
PAED: Pediatric anesthesia emergence delirium
PONV: Postoperative nausea and vomiting
SD: Standard deviation
IQR: Interquartile range
